# PIS-Net: Efficient Medical Image Segmentation Network with Multivariate Downsampling for Point-of-Care

**DOI:** 10.3390/e26040284

**Published:** 2024-03-26

**Authors:** Changrui Zhang, Jia Wang

**Affiliations:** School of Computer Science and Technology, Xinjiang University, Urumqi 830046, China; 107552103569@stu.xju.edu.cn

**Keywords:** point-of-care (PoC), medical image segmentation, efficient downsampling strategy, MLP–CNN hybrid model, image distortion

## Abstract

Recently, with more portable diagnostic devices being moved to people anywhere, point-of-care (PoC) imaging has become more convenient and more popular than the traditional “bed imaging”. Instant image segmentation, as an important technology of computer vision, is receiving more and more attention in PoC diagnosis. However, the image distortion caused by image preprocessing and the low resolution of medical images extracted by PoC devices are urgent problems that need to be solved. Moreover, more efficient feature representation is necessary in the design of instant image segmentation. In this paper, a new feature representation considering the relationships among local features with minimal parameters and a lower computational complexity is proposed. Since a feature window sliding along a diagonal can capture more pluralistic features, a Diagonal-Axial Multi-Layer Perceptron is designed to obtain the global correlation among local features for a more comprehensive feature representation. Additionally, a new multi-scale feature fusion is proposed to integrate nonlinear features with linear ones to obtain a more precise feature representation. Richer features are figured out. In order to improve the generalization of the models, a dynamic residual spatial pyramid pooling based on various receptive fields is constructed according to different sizes of images, which alleviates the influence of image distortion. The experimental results show that the proposed strategy has better performance on instant image segmentation. Notably, it yields an average improvement of 1.31% in Dice than existing strategies on the BUSI, ISIC2018 and MoNuSeg datasets.

## 1. Introduction

In recent years, the development of portable medical imaging devices has created great potential for point-of-care (PoC) [1,2]. In other words, medical imaging services are shepherded to places near to people such as pharmacies, gyms and supermarkets. According to the U.S. Centers for Disease Control and Prevention (CDC) [3], the data from ongoing imaging studies are growing by as much as 5% per year, while the number of radiologists grows by only about 2% per year. Therefore, the development of image analysis by AI is urgently needed to advance PoC. Instant image segmentation, as one of the most important tasks in image analysis, is thus becoming a research hotpot.

**Figure 1:**
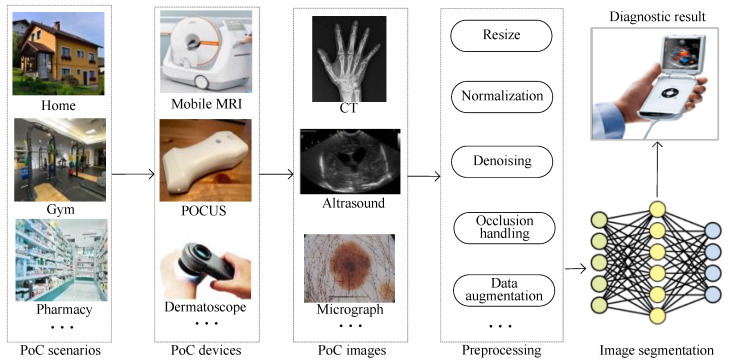
An illustration of PoC image solutions based on image segmentation.

In general, different portable PoC devices generate medical images with different sizes, as shown in Figure 1. These medical images need preprocessing by resizing, normalization, data augmentation and so on. In other words, image distortion due to preprocessing is inevitable. The distortion of the image leads to blurred edges and inconspicuous foreground–background differences, which reduces the effectiveness of the semantic segmentation task. After preprocessing, images are fed into deep learning models for image segmentation. The pixels of these distorted images always interact with each other. And the complex diverse features are difficult to extract. Without doubt, a more efficient feature representation for distorted data needs to be designed. The instant image segmentation diagnosis results are shown on portable devices. With the advantages of strong feature extraction, a small number of parameters and fast inference, MLP–CNN hybrid models [4] have better instant segmentation ability and thus have more potential to be applied in PoC scenarios.

**Figure 2:**
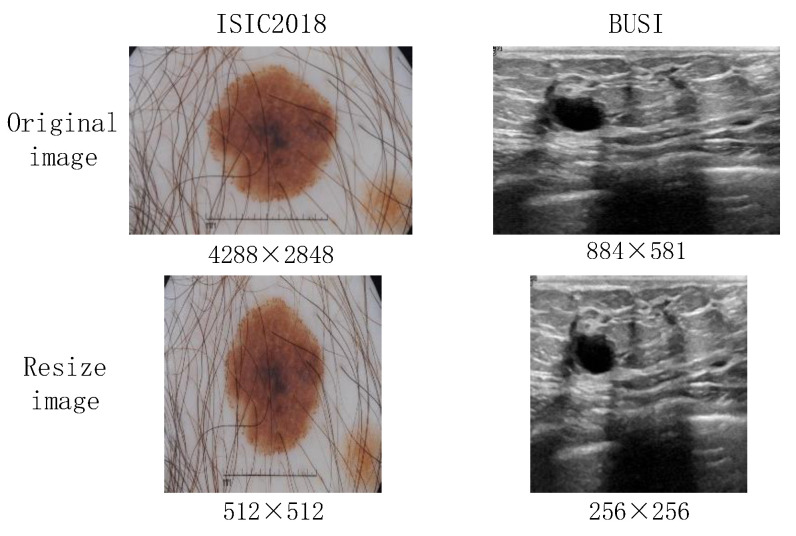
Image distortion after resizing for ISIC2018 [5,6] and BUSI [7].

Generally, medical images are characterized by having complex and diverse features. The existing downsampling structures of hybrid models are too single to make it difficult to learn sufficient feature representations. Moreover, the preprocessing results in image distortion, which is shown in Figure 2. Existing hybrid models frequently use a linear layer in a Multilayer Perceptron (MLP) block. In other words, image distortion is particularly severe in MLP–CNN hybrid models.

In this paper, a PoC image segmentation network (PIS-Net) is proposed to obtain a richer feature representation and reduce the influences of image distortion. In this network, we design a new Diagonal-Axial MLP Block to extract rich global features by utilizing parallel diagonally oriented sliding windows with connections between neighboring windows. Additionally, a hybrid downsampling strategy (HBDS) is figured out to fuse multi-scale features by complementing the linear features extracted by MLP with nonlinear features. In addition, our proposed Dynamic Residual Spatial Pyramid Pooling (DR-SPP) can adaptively enrich the respective field of the feature set extracted in the downsampling stage to reduce the influences of image distortion and simplify the network structure. The main contributions of this paper are as follows:

1. We propose a Diagonal-Axial MLP as a new MLP module with windows on the diagonal axis, which uses parallel sliding windows in dual diagonal directions with partial overlap between the windows. The module sufficiently enhances the feature interactions between individual sliding windows in the channel dimension to achieve the effect of modeling global long-range dependencies, which guarantees good local feature extraction to obtain richer semantic features.
2. In order to enhance the semantic and geometric information representation capabilities, we propose an HBDS. In the MLP stage of downsampling, we add a mixed downsampling branch to supplement the linear features with nonlinear features to achieve the effect of multi-scale feature fusion.
3. Inspired by the parallel pooling layers in spatial pyramid pooling (SPP) [8], we construct a DR-SPP to provide various receptive fields with parallel pooling layers for the upsampling stage, which mitigates the image distortion of the MLP–CNN hybrid models.

The rest of the paper is organized as follows: Section 2 briefly describes the related work of deep learning based on image segmentation. In Section 3, details of the PIS-Net are given. Section 4 presents the experimental results, and Section 5 discusses some directions related to PIS-Net. Our conclusion and future work are shown in Section 6.

## 2. Related Works

In terms of different baselines of image segmentation based on deep learning [9,10], existing methods can be classified in CNN-based models [11], transformer-based models [12] and hybrid models.

### 2.1. CNN-Based Models

In the field of computer medicine, image segmentation methods based on convolutional neural networks (CNNs) initially achieved advanced results. Long et al. [13] proposed an FCN network consisting of convolutional and pooling layers with skip connections, which had the advantage of accepting images of arbitrary sizes. Since the single upsampling cannot yield clear segmentation results, Ronneberger et al. [14] introduced the U-Net network based on the FCN, which achieves more accurate predictions by incorporating additional convolutional layers during upsampling. In recent years, researchers have made significant breakthroughs by using U-Net as a baseline method. The U-Net++ model [15] combines long and short skip connections to integrate features from different levels through feature concatenation, which improves feature fusion. ResU-Net [16] incorporates residual connections to alleviate gradient vanishing and enhances the integration of contextual information. U-Net3+ [17] introduces full-scale skip connections to facilitate the combination of low-level details and high-level semantics from different-scale feature maps to enhance segmentation accuracy. To construct lightweight segmentation models, DSNet [18] and Separable-U-Net [19] replace traditional convolutions with depthwise separable convolutions (DWConv), which effectively reduce the number of model parameters.

Although these CNN-based methods have achieved great success, due to the localization of the convolution and the translation invariance, CNN-based models lack the learning of global information when performing segmentation of complex images with high requirements (e.g., medical images and remote sensing images).

### 2.2. Transformer-Based Models

Originally, transformer-based models were designed to solve natural language processing (NLP) tasks [20,21]. Now, these models are widely used in machine translation, text mining and other fields. Dosovitskiy [22] et al. adapted the transformer model to the field of computer vision and proposed the Vision Transformer (ViT), which solves the problem of the long input sequence and the complex computation of the models. TransU-Net [23] modifies the ViT architecture into a U-shaped network for 2D medical image segmentation. It encodes the tokenized image blocks in the CNN feature mapping to extract a global context to solve the problem of the limitation of remote dependency. MedT [24] introduces an additional control mechanism for the self-attention module, along with a two-branch architecture for learning both global and local features. TransBTS [25] uses 3D CNN to extract 3D spatial features and input them into a transformer model for global feature modeling. A progressive upsampling is performed to eventually obtain a detailed segmentation map. UNETR [26] formulates the task of 3D medical image segmentation as a sequence-to-sequence prediction problem and strengthens the interaction between the encoder and decoder through powerful skip connections to efficiently capture global multi-scale information.

Though the aforementioned transformer-based approaches have achieved highly advanced levels of accuracy, they often suffer from issues such as excessively high computational complexity, lengthy inference times and a large number of parameters. These challenges can significantly hinder the practical application of these models in PoC (point-of-care) scenarios.

### 2.3. Hybrid Models

In order to make full use of advantages of model research, hybrid models are mainly aimed at combining different features of different models and are applied to image segmentation. The main two hybrid models are the combining of CNN and transformer features and CNN and MLP features for image segmentation. CNN–Transformer hybrid models have the advantage of combining the feature extraction capabilities of CNNs with the long-range dependency modeling capabilities of transformers. LET-Net [27] combines a U-shaped CNN with a transformer effectively in a capsule embedding style to compensate for respective deficiencies; Yuan et al. [28] proposed CTC-Net, which utilizes dual coding paths of CNNs and transformer encoders to produce complementary features. ScribFormer [29] improves model performance by utilizing a three-branch structure to unify shallow and deep features, which consists of a mixture of CNN branches, transformer branches, and attention-guided class activation map (ACAM) branches. CNN-MLP hybrid models’ strength is that it can achieve better segmentation results while having a small number of parameters and fast reasoning. UNeXt [4] introduces the sliding window idea of the Swin Transformer [30] and designs the Tok-MLP module, which effectively enhances the model’s ability to capture long-distance dependencies between features and achieves advanced performance on the ISIC2018 and BUSI datasets with low consumption. The subsequent works based on this foundation include G-UNeXt [31] and Res2-UNeXt [32]. The former uses linear computation to reduce the computational cost of redundant features, and the latter enhances the fusion of multi-scale information.

These studies showed that the multi-stage mixed modeling framework consistently and significantly outperformed the single-stage framework. Relative to the CNN–Transformer hybrid model, the immediacy of MLP–CNN hybrid models offers more potential for PoC scenarios. Nevertheless, the homogeneous downsampling of MLP–CNN hybrid models cannot capture enough features for complex image segmentation. Compared to the existing models for image segmentation, two different aspects are considered in this paper: (1) For instant image segmentation, medical images always contain complex features and diverse details. Existing MLP–CNN hybrid models cannot learn richer and more global features for the robustness and generalization of models. (2) Various medical images must always be resized for fixed-size image segmentation, while image distortion emerges and has great influences on image segmentation. In this paper, we propose PIS-Net to obtain more global features and implement results by image distortion.

## 3. Methodology

### 3.1. Overall Network Architecture

**Figure 3:**
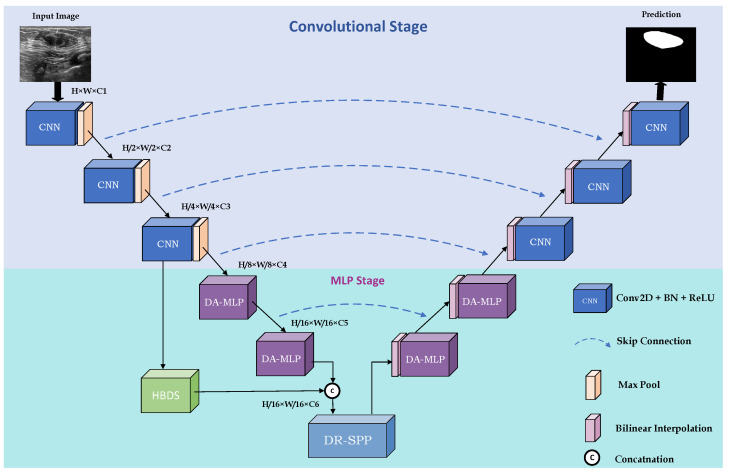
Overall structure of the proposed PIS-Net.

The overall architecture of PIS-Net is shown in Figure 3. PIS-Net is a multi-stage network based on an encoder and decoder, including a CNN stage and MLP stage. The CNN stage consists of conventional CNN blocks stacking together for powerful feature extraction capabilities. The MLP stage is mainly composed of our proposed Diagonal-Axial MLP Block, which is different from the existing MLP block. We design a set of parallel shift windows in the diagonal direction, which solves the problem of the single representation of features extracted by the existing CNN–MLP hybrid models. Notably, the Diagonal-Axial MLP Block has no increment of the number of parameters, especially when facing complex medical images. To improve the model’s generalization ability, we use HBDS in the MLP stage to extract rich multi-scale features. To address the serious distortion problem of medical images, we insert DR-SPP at the end of downsampling to give the feature set more diverse receptive fields and improve the overall robustness.

### 3.2. Diagonal-Axial MLP Block (DA-MLP)

It is well known that windowing the feature set is an effective way to address the model training cost, which has been studied in convolutional modules and attention modules. However, the existing sliding window-based MLP blocks are not considered the establishment of interactions between individual local windows in different channel dimensions, which leads to the loss of global information and reduces the effectiveness of modeling global long-range dependencies. In this paper, we propose the Diagonal-Axial MLP Block to enhance the information interaction between the windows. We design a new parallel sliding window in dual diagonal directions to achieve the effect of modeling global long-range dependencies.

**Figure 4:**
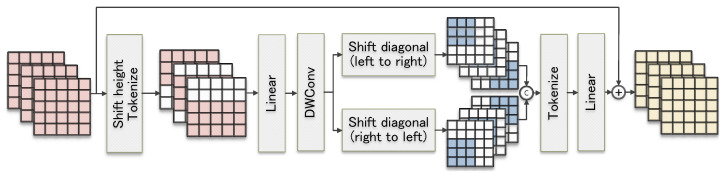
Structure of the Diagonal-Axial MLP Block.

The internal structure of the Diagonal-Axial MLP Block is shown in Figure 4. Overall, the structure of the Diagonal-Axial MLP Block is a three-layer network formed by inserting a depthwise convolution (DWConv) layer between two of the shifted linear layers.

The first shifted linear layer shifts the input features in height in the usual way, which introduces window-based localized features to the regular MLP block. Then, the features are projected into tokens and sent to the linear layer. Suppose that the input feature is $X_{in}$ and that $X_{h}$ is the output feature of the first shifted linear layer. The process can be simply executed as

(1) $$\begin{array}{c} X_{h}=f_{Linear}(Tok\left(Shift_{h}\left(X_{in}\right)\right) \end{array}$$

where $Shift_{h}$ denotes the routine method of $X_{in}$ shifting in height and Tok denotes the projection of the features into tokens.

After obtaining $X_{h}$, it passes through a DWConv [33] layer; this can encode the position information [33] for $X_{h}$ to obtain a more diversified set of features with a small number of parameters, which we denote by $X_{m}$. The process can be simply executed as

(2) $$\begin{array}{c} X_{m}=f_{DWConv}\left(X_{h}\right) \end{array}$$

Since the extracted feature representations from the independent conventional windows are more homogeneous, they are not favorable for global feature extraction, and there are redundant computations when applied to medical images with an edge-independent nature. Therefore, we design a new training method with shifted windows in the second shifted linear layer. First, a two-branch parallel training method is used, where the shift directions of the windows in different branches are various, which realizes that the extraction of richer feature representations can not increase the number of parameters. Moreover, the shift distance of the windows is half of the window size, which causes the windows to partially overlap with each other to establish a connection between the windows. Additionally, it can realize the transfer of parameters between the windows and strengthen the model’s learning of global information.

Assuming that the dimension of the current input feature $X_{m}$ is (C,H,W), the size of the shift window is denoted by $shift_{size}$. And $shift_{size}$ will adaptively take the value $shift_{size}=\left\lfloor \sqrt[{4}]{H\cdot W}\right\rfloor$. To maintain dimensional consistency, an edge padding operation of $padding_{size}=\frac{\left\lfloor \sqrt[{4}]{H\cdot W}\right\rfloor }{2}$ is performed on $X_{m}$. After the shifting is complete, Concat is performed on the channel dimension with the feature set obtained on all branches to yield the feature set $X_{d}$ of dimension (2C,H,W). Eventually, $X_{d}$ is fed into the linear layer for training, and the training results are linearly summed with $X_{in}$, which results in a feature set $X_{out}$ containing rich feature representations. Assuming that $Shift_{lr}$ represents the operation of moving the window from the bottom-left corner to the top-right corner and that $Shift_{rl}$ represents the operation of shifting the window from the bottom-right corner to the top-left corner, the following equation accurately describes the process:

(3) $$\begin{array}{c} X_{d}=Tok(f_{Concat}\left(Shift_{lr}\left(X_{m}\right),Shift_{rl}\left(X_{m}\right)\right) \end{array}$$

(4) $$\begin{array}{c} X_{out}=X_{in}+f_{Linear}\left(X_{d}\right) \end{array}$$

### 3.3. Hybrid Downsampling Strategy (HBDS)

**Figure 5:**
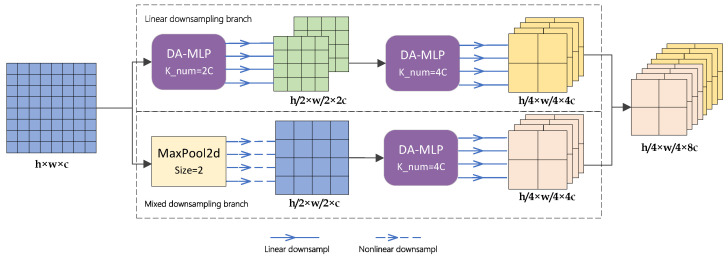
Flow of the hybrid downsampling strategy, where *K_num* represents the number of kernels in the depthwise separable convolution layer within the DA-MLP block.

In order to achieve multi-scale fusion, most deep-learning-based network models involve the simple matrix splicing of features with different depths, which results in the feature set containing many shallow and redundant features in the downsampling stage. In this paper, we propose a hybrid downsampling strategy for the downsampling stage to enhance the semantic and geometric information representation capabilities. Moreover, more efficient multi-scale fusion with fewer parameters is obtained. Different from the common multi-scale fusion methods, our proposed hybrid downsampling strategy aims to fuse linear features with nonlinear features by designing a mixed downsampling branch to supplement nonlinear features to a single linear downsampling branch. The specific process is shown in Figure 5.

Assume that the input feature map to the MLP stage is denoted as $F_{in}$ with dimensions h×w×c. After passing through the original linear downsampling branch, $F_{in}$ will be linearly downsampled twice to update the feature parameters. The output of the linear downsampling generates a set of features denoted as $F_{L}$ with dimensions h/4×w/4×4c. Simultaneously, $F_{in}$ enters the mixed downsampling branch. $F_{in}$ first undergoes a MaxPool layer with a pool size of 2 to divide $F_{in}$ into several 2×2 squares. Within each square, the maximum feature value is retained only, while the original spatial arrangement is maintained. This process yields a nonlinear feature collection denoted as $F_{in}^{\prime }$. Subsequently, $F_{in}^{\prime }$ is input into a DA-MLP block with a kernel number of 4. This block performs linear learning on $F_{in}^{\prime }$ to produce a feature set denoted as $F_{M}$ of dimensions h/4×w/4×4c, which combines linear and nonlinear features.

The concatenation operation is performed on the channel dimension between $F_{L}$ and $F_{M}$. The downsampling stage yields a feature set $F_{out}$ that is enriched in both semantic and geometric information. The above process can be executed as

(5) $$\begin{array}{c} F_{L}=f_{DA-MLPs}\left(f_{DA-MLPs}\left(F_{in}\right)\right) \end{array}$$

(6) $$\begin{array}{c} F_{M}=f_{DA-MLPl}\left(f_{MaxPool}\left(F_{in}\right)\right) \end{array}$$

(7) $$\begin{array}{c} F_{out}=f_{Concat}\left(F_{L},F_{M}\right) \end{array}$$

DA-MLPs and DA-MLPl represent DA-MLP blocks with ratios of the number of input channels to the number of output channels of 1:2 and 1:4, respectively.

### 3.4. Dynamic Residual Spatial Pyramid Pooling Block (DR-SPP)

In the field of computer vision, the problem of image distortion due to resizing has always been a key factor affecting the performance of models, especially in instant image segmentation. Inspired by the spatial pyramid pooling layer and its variants [34], we propose the more efficient DR-SPP Block to alleviate the image distortion of CNN-MLP hybrid models for the first time.

**Figure 6:**
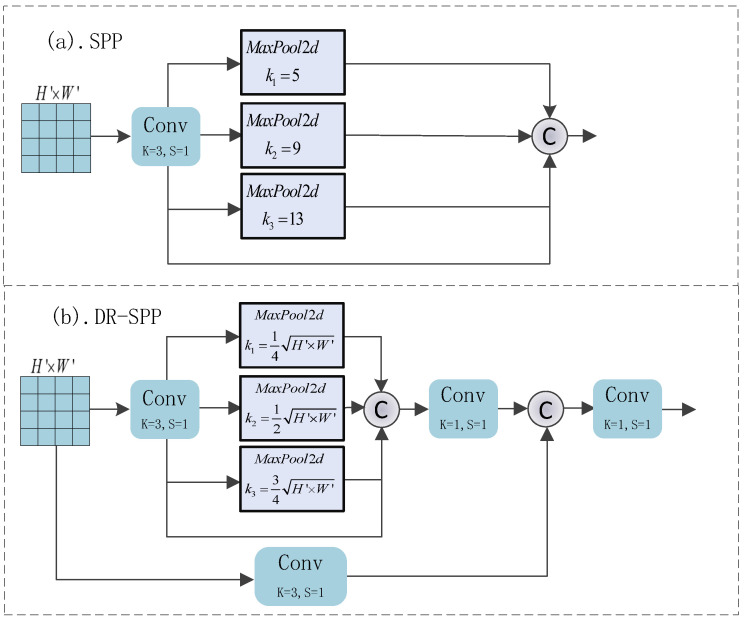
Structures of SPP and DR-SPP are shown in (**a**) and (**b**), respectively.

The specific structures of SPP and DR-SPP are shown in Figure 6. Among them, a parallel multi-scale pooling layer can give the feature set a richer receptive field, thus enabling the model to adapt to the change in image dimensions and solving the problem of edge blurring and foreground–background difference reduction caused by image distortion. Moreover, our proposed DR-SPP can adaptively select the size of the pooling window according to the dimensions of the input features; the addition of parallel convolutional residual branches can render the pooling process more stable and reduce the fluctuations in weights.

Assuming that the size of the feature set input to the DR-SPP module is $H^{\prime }\times W^{\prime }\times C^{\prime }$, the pooling kernel size of MaxPool2d is adaptively valued according to the input feature set, i.e., $k_{i}=\frac{i\times \sqrt{H^{\prime }\cdot W^{\prime }}}{4},i\in \left\{1,2,3\right\}$. *i* is the level of pooling in DR-SPP. The value of padding is adaptively set according to the pooling kernel size, i.e., $p=\frac{k_{i}}{2},i\in \left\{1,2,3\right\}$. After passing through the parallel MaxPool unit, the input feature maps will, respectively, output a feature set $F_{i}$ of size $\frac{H^{\prime }}{k_{i}}\times \frac{W^{\prime }}{k_{i}}\times C^{\prime },i\in \left\{1,2,3\right\}$. The feature set concatenates $F_{i}$ in the channel dimensions; they are fed into a convolutional layer for fusion, which yields $dim=4C^{\prime }$ and contains a feature set with multiple receptive fields. Through the above steps, we ensure that, regardless of the resolution of the input image before resizing, the final DR-SPP can effectively mitigate the loss of key information due to the distortion of the image, ensuring the robustness of the model. Additionally, the pooling layer does not need to optimize the parameters, which reduces the risk of model overfitting due to the excessive number of layers.

### 3.5. Loss Function

Since the loss function can optimize the model by closing the gap between the predicted and true values, selecting a loss function that fits the model can yield better results. In this work, we choose the combination of binary cross-entropy (BCE) and Dice loss to train PIS-NET [35]. The computational procedure is defined as follows:

(8) $$\begin{array}{c} L_{BCE}=-\frac{1}{N}\sum_{i}^{N}\left(t_{i}\log o_{i}+\left(1-t_{i}\right)\log\left(1-o_{i}\right)\right) \end{array}$$

(9) $$\begin{array}{c} L_{\mathrm{Dice}\;}=1-2\times \frac{\sum_{i}^{N}o_{i}t_{i}}{\sum_{i}^{N}o_{i}+\sum_{i}^{N}t_{i}} \end{array}$$

(10) $$\begin{array}{c} L=\lambda_{1}L_{BCE}+\lambda_{2}L_{\mathrm{Dice}\;} \end{array}$$

where *i* represents the index of all pixels, $o_{i}$ is the probability map and $t_{i}$ is the ground truth annotation. We denote the total number of pixels by *N*. Finally, the coefficients $\lambda_{1}$ and $\lambda_{2}$ are empirically set as 0.5 and 1, respectively.

## 4. Experiments and Result Analysis

### 4.1. Datasets and Preprocessing

In order to fully evaluate the performance of the model, we selected the Breast Ultrasound Images (BUSI), International Skin Imaging Collaboration (ISIC2018) and Multi-Organ Kernel Segmentation (MoNuSeg 2018) [36,37] datasets as benchmarks.

1. BUSI: This dataset is derived from 600 female breast cancer patients. The images have an average pixel size of 500 × 500. To ensure experimental fairness, similar to the baseline model, only samples of the categories benign and malignant are used, resulting in a total of 647 images with a resized resolution of 256 × 256. The dataset is characterized by a small sample size, and the ultrasound images therein exhibit low contrast and uneven grayscale, leading to the presence of a substantial amount of noise.
2. ISIC2018: This dataset consists of skin images containing cases and corresponding segmentation images of skin lesions, including a total of 2594 images. We resize all images to 512 × 512. The images have a relatively high resolution, which leads to significant distortion after resizing.
3. MoNuSeg 2018: The training data comprise 30 images labeled with approximately 22,000 nuclear boundaries, and the test data comprise 14 images labeled with more than 7000 nuclear boundaries. In the experiment, we slice the sample images and finally obtain 1080 training set and 504 validation set images with a resolution of 256 × 256. This dataset has a diversity of characteristics because it is collected from different organs in multiple patients; at the same time, there are large differences in the size of the cells.

In conclusion, conducting experimental comparisons on these three datasets with distinct characteristics allows a comprehensive assessment of the model’s performance and validates the generalization capability of our model across diverse medical image segmentation tasks.

### 4.2. Comparison Methods

The principles guiding the selection of comparison methods are based on the reproducibility principle. Apart from our baseline model, in this paper, we aim to select the latest open-source models. The chosen methods are all considered classic and representative within the medical image segmentation field. They encompass convolutional baselines such as U-Net [14], U-Net++ [15], U-Net3+ [17] and ResU-Net [16]; recent transformer-based baselines such as TransU-Net [23] and MedT [24]; the CNN–Transformer hybrid model ScribFormer [29]; and the CNN–MLP hybrid model UNeXt [4].

Please note that the criteria used to assess the performance of our model encompass segmentation metrics (Dice and IoU), the parameter count, the computational complexity (measured in GFLOPs) and the inference time (measured in milliseconds), among others. Due to the randomness associated with dataset partitioning, to ensure experimental fairness, we re-conducted all comparison method experiments on the three datasets. All experiments were conducted in the same hardware and software environment, with experimental settings consistent with those described in the original paper. The expressions for the quantitative metrics are as follows:

(11) $$\begin{array}{c} \mathrm{IoU}=\frac{\mathrm{TP}}{\mathrm{TP}+\mathrm{FP}+\mathrm{FN}} \end{array}$$

(12) $$\begin{array}{c} \;\mathrm{Dice}\;=\frac{2\mathrm{TP}}{\mathrm{FP}+2\mathrm{TP}+\mathrm{FN}} \end{array}$$

where TP denotes true positives, indicating a correctly predicted change pixel; FP denotes false positives, indicating an incorrectly predicted change pixel; and FN denotes false negatives, indicating an incorrectly predicted unchanged pixel.

### 4.3. Implementation Details

We developed PIS-Net using the Pytorch framework. For the optimizer, we chose Adam with a learning rate of 0.0001 and momentum of 0.9. We also used a cosine annealing learning rate scheduler with a minimum learning rate of 0.00001. The batch size was set to 8. The PIS-Net model was trained for a total of 400 epochs. We divided the dataset into training and test sets at a ratio of 8:2, and we fixed the randomization of the division of the dataset to ensure that all experiments were conducted on the same training and test sets. All experiments were performed on RTX A5000 GPUs.

### 4.4. Comparative Experiments

#### 4.4.1. Quantitative Analysis of the Results of Comparative Experiments

The results of the quantitative comparison experiments of the different methods on the three datasets are shown in Table 1.

Among the CNN-based models, U-Net++, ResU-Net and U-Net3+, all show a small improvement in the Dice and IoU metrics compared to U-Net. However, U-Net++ has a great advantage in terms of the number of parameters compared to the other CNN models due to its flexible network structure, which enables the branching and trimming of the model, with only 9.16 M. In the transformer-based model, MedT, although it has only 1.6 M parameters, it does not show a significant improvement in the Dice and IoU metrics; due to its use of a special sparse attention mechanism, the inference speed is also slower. Moreover, TransU-Net has a significant improvement in the Dice and IoU metrics compared to the traditional U-Net model and MedT, but the number of parameters reaches 105.32M, which is equivalent to 20 times that of our model. ScribFormer has significant advantages in terms of segmentation accuracy, but due to its three-branch structure, it has too many parameters and is not suitable for PoC scenarios. In the hybrid CNN–MLP-based model, UNeXt and PIS-Net (ours) achieve high Dice and IoU metrics while maintaining a small number of parameters and GFLOPs; however, it is worth noting that the improvement that we made in the PIS-Net model fails to affect the model’s inference speed, while outperforming all the comparative models, improving the IoU by 1.04%, 0.43% and 3.16%, vs. the Dice by 2.17%, 0.61% and 1.15% compared to UNeXt on the BUSI, ISIC2018 and MoNuSeg datasets, respectively.

#### 4.4.2. Visualization Analysis on BUSI

**Figure 7:**
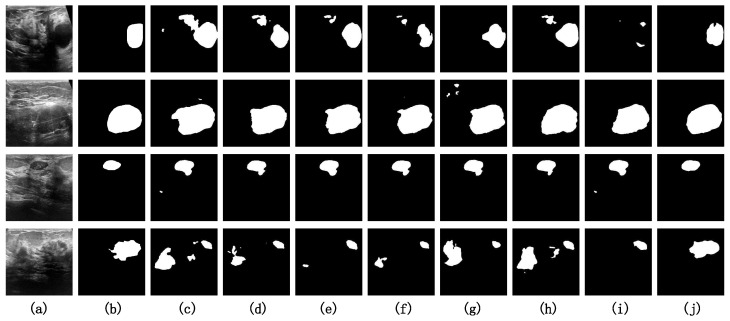
Visualization results on the BUSI dataset. (**a**) Original Image. (**b**) Ground Truth. (**c**) U-Net. (**d**) U-Net++. (**e**) ResU-Net. (**f**) U-Net3+. (**g**) MedT. (**h**) TransU-Net. (**i**) UNeXt. (**j**) PIS-Net (ours).

Figure 7 presents the visual results of comparative experiments on several challenging samples from the BUSI dataset. Observing the source images of the samples shows that effectively teaching the model to distinguish between noise and the target is the primary challenge of the BUSI dataset in the context of semantic segmentation tasks. We have indicated the areas in the source images that are prone to incorrect identification as true positives—these are the noisy regions—using red circles. For the first three samples, all models except ours misidentify the noisy regions as target regions to varying extents. In the case of the fourth sample, only our model accurately recognizes the target region while circumventing the interference caused by the noise.

#### 4.4.3. Visualization Analysis on ISIC2018

**Figure 8:**
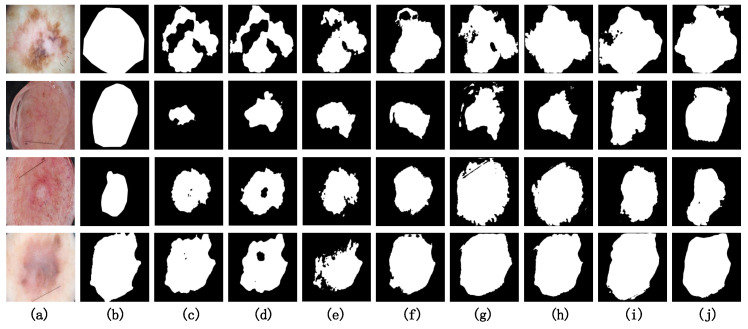
Visualization results on the ISIC2018 dataset. (**a**) Original Image. (**b**) Ground Truth. (**c**) U-Net. (**d**) U-Net++. (**e**) ResU-Net. (**f**) U-Net3+. (**g**) MedT. (**h**) TransU-Net. (**i**) UNeXt. (**j**) PIS-Net (ours).

Figure 8 presents the visual results of the comparative experiments on several challenging samples from the ISIC2018 dataset. Due to the high resolution of the ISIC2018 dataset and the significant variations in the aspect ratio among many samples, image distortion poses a pronounced challenge in semantic segmentation tasks. Among the CNN-based models (c, d, e and f), the segmentation results for these four challenging samples are less satisfactory, with notable instances of under-segmentation in comparison to other models. It is worth noting that our model (j) achieves segmentation results along the edges of the target regions that closely resemble the ground truth (b). This is attributed to the shifting MLP window within the Diagonal-Axial MLP Block, which allows the model to effectively learn the relative positional information between features.

#### 4.4.4. Visualization Analysis on MoNuSeg

**Figure 9:**
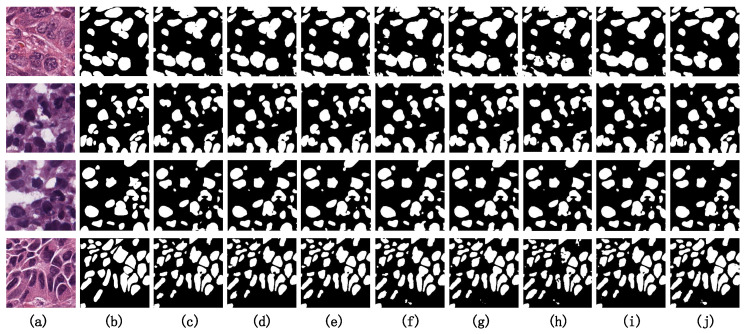
Visualization results on the MoNuSeg dataset. (**a**) Original Image. (**b**) Ground Truth. (**c**) U-Net. (**d**) U-Net++. (**e**) ResU-Net. (**f**) U-Net3+. (**g**) MedT. (**h**) TransU-Net. (**i**) UNeXt. (**j**) PIS-Net (ours).

Figure 9 presents the visual results of the comparative experiments on several challenging samples from the MoNuSeg dataset. By observing the original image, we can conclude that a significant challenge in the semantic segmentation task of the MoNuSeg dataset is achieving the accurate segmentation of small objects. For ease of comparison, we have marked the difficult-to-segment small object regions with red circles. Among all the model visualization results, PIS-Net demonstrates generally superior segmentation performance compared to the other benchmark models. However, in the case of the third sample, there is an issue of missed detection, indicating that our model’s precision in segmenting faint small objects is not yet optimal. Enhancing the accuracy in the segmentation of such faint small objects is a critical area for improvement in our model.

### 4.5. Comparative Experiments

In order to verify the effects of the Diagonal-Axial MLP Block, DR-SPP Block and hybrid downsampling strategy introduced during the model design process on the model performance, we designed the following ablation experiments, which are referred to as Experiment 1 to Experiment 6.

1. U-Net: The original U-Net with 64 channels in the first stage.
2. Conv Stage + Tok-MLP (UNeXt): The baseline model, built upon the U-Net architecture, which replaces the fourth and fifth stages of the network with Tok-MLP blocks.
3. Conv Stage + Diagonal-Axial MLP: Built upon the UNeXt architecture, the fourth and fifth stages of the network are replaced by the Diagonal-Axial MLP Block proposed by us.
4. Conv Stage + Diagonal-Axial MLP + DR-SPP: While incorporating the Diagonal-Axial MLP Block, the model includes the insertion of the DR-SPP Block after the downsampling process.
5. Conv Stage + Diagonal-Axial MLP + HBDS: While integrating the Diagonal-Axial MLP Block, the model also employs the hybrid downsampling strategy, wherein a parallel HBDS Block is introduced during the MLP stage of the downsampling process.
6. Conv Stage + Diagonal-Axial MLP + DR-SPP + HBDS (PIS-Net): This is our model, PIS-Net, after adding all the improvements.

The results of the ablation experiments are shown in Table 2.

**Figure 10:**
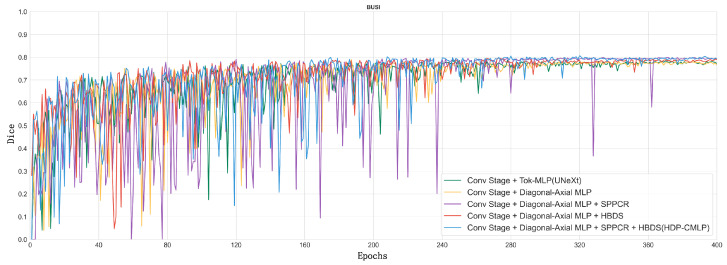
Dice curves for the validation set of each ablation experiment model on the BUSI dataset.

In order to visualize the model’s “evolution” process, we show in Figure 10 a line plot of the Dice metrics of the baseline model (2. UNeXt) and the ablation model on the validation set when training on the BUSI dataset.

**Figure 11:**
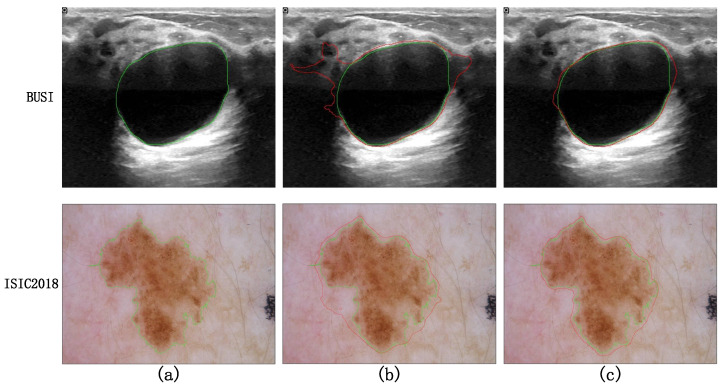
Visualization results of Experiment 5 and Experiment 6. Among them, the green line in (**a**) represents the ground truth. (**b**) The red lines represent the results of Experiment 5, and the green lines represent the ground truth. (**c**) The red lines represent the results of Experiment 6, and the green lines represent the ground truth.

Taking a comprehensive analysis of Table 2 and Figure 10 into account, it can be observed that the MLP phase of Experiment 1, employing parallel diagonal-axial sliding windows, enhances the inter-window feature interaction, effectively capturing global long-range dependencies. This results in a 0.52 improvement in the Dice metric compared to UNeXt, and the Dice curve converges to stability earlier. Upon the incorporation of the DR-SPP module, image distortion issues are addressed, leading to a noticeable enhancement in the Dice metric. The visualization results of Experiment 5 and Experiment 6 on difficult samples are shown in Figure 11. After adding DR-SPP, the model can better fit the edges of the target area and more accurately identify the foreground and background. However, due to the broader receptive field of the feature set generated during the downsampling phase, the Dice curve exhibits greater fluctuations during training.

Experiment 5 utilizes a mixed downsampling strategy in the downsampling phase, supplementing nonlinear downsampling features with the linear ones from the MLP phase, effectively achieving multi-scale feature fusion. This results in a significant improvement in the Dice metric, while the training process remains relatively stable.

Finally, our “complete body” model, which combines all methods, achieves a 2.17 increase in the Dice metric compared to the baseline model. The Dice curve is smoother and converges earlier, showcasing advanced performance in semantic segmentation on the BUSI dataset.

## 5. Discussion

**Figure 12:**
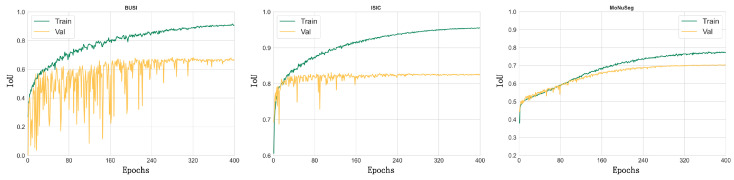
The IoU curves of the PIS-Net model on the BUSI, ISIC and MoNuSeg datasets, with green representing the training set and yellow the validation set.

We show the IoU curves of the PIS-Net model on the three datasets for the training set compared with the validation set in Figure 12, respectively. A comprehensive analysis reveals that our model has achieved excellent performance on these three datasets, even with the small sample sizes of the instant medical datasets. From Figure 12, the convergence of our model on BUSI is lower. The reason lies in that more noise is presented in the BUSI dataset than in the ISIC and MoNuSeg datasets. The IoU curves of our model for the BUSI dataset show that the noise problem is more serious compared to the ISIC and MoNuSeg datasets. In conclusion, the main areas for improvement in the PIS-Net model are to further enhance the robustness on small-sample datasets and to further overcome the influence of noise on the training process.

## 6. Conclusions and Future Work

In this work, we propose an MLP–CNN hybrid model, PIS-Net, for medical instant image segmentation with low resolution. In the MLP phase of the model, we propose the Diagonal-Axial MLP Block to focus on local window attention by parallel diagonal-axial sliding windows to enhance the information interactions among windows. The designed Diagonal-Axial MLP Block also can achieve the effect of modeling global long-range dependencies. At the end of the downsampling stage, the DR-SPP module with parallel pooling is used to adaptively select the size of the pooling window according to various dimensions of the input features. DR-SPP is inserted to obtain a richer output feature map with a richer receptive field for model upsampling, which alleviates the image distortion by preprocessing. The figured HBDS utilizes the complementarity of nonlinear downsampling and linear downsampling to achieve the effect of multi-scale feature fusion for a better feature representation. Our experiments on three public datasets show that PIS-Net can obtain advanced performance metric scores (IoU and Dice), while the number of model parameters is small and the training speed is fast. However, PIS-Net shows a certain degree of overfitting on datasets with small sample sizes. Our future work will aim to propose data augmentation and transfer learning methods for PIS-Net.

## Figures and Tables

**Table 1:** Comparative experimental results for the three datasets.

Method	Params (in M)	Inference Speed (in ms)	GFLOPs	BUSI		ISIC2018		MoNuSeg	
				IoU	Dice	IoU	Dice	IoU	Dice
U-Net	31.13	223	55.84	63.85	76.35	74.55	84.03	65.99	79.43
U-Net++	9.16	173	34.65	64.33	77.54	75.12	84.96	66.04	79.49
ResU-Net	62.74	333	94.56	64.89	77.71	75.62	85.60	66.07	79.50
U-Net3+	26.97	197	48.26	65.33	77.73	78.66	87.81	66.56	72.16
MedT	1.6	751	21.24	63.89	76.93	79.54	87.35	62.49	76.83
TransU-Net	105.32	246	38.52	66.81	78.34	80.51	88.91	66.17	79.55
ScribFormer	50.44	532	37.63	67.59	78.69	81.96	89.93	67.36	81.29
UNeXt	1.47	25	0.57	67.24	78.49	82.25	90.15	67.24	81.36
PIS-Net (ours)	5.46	29	0.82	68.28	80.66	82.68	90.76	70.40	82.51

**Table 2:** Quantitative results of the model ablation experiments on the BUSI and ISIC2018 datasets.

Method	Params (in M)	Inf. Time (in ms)	BUSI		ISIC2018	
			IoU	Dice	IoU	Dice
1. Original U-Net	31.19	223	63.85	76.35	74.55	84.03
2. Conv Stage + Tok-MLP (UNeXt)	1.47	25	65.86	78.49	82.05	90.15
3. Conv Stage + Diagonal-Axial MLP	1.61	27	65.99	79.01	82.31	90.32
4. Conv Stage + Diagonal-Axial MLP + DR-SPP	4.75	39	67.28	79.90	82.57	90.63
5. Conv Stage + Diagonal-Axial MLP + HBDS	1.78	27	66.71	79.65	82.49	90.54
6. Conv Stage + Diagonal-Axial MLP + DR-SPP + HBDS (ours)	5.46	29	68.28	80.66	82.68	90.76

## Data Availability

The subject data used in this study were obtained from the following datasets: the International Skin Imaging Collaboration (ISIC2018) database (https://challenge.isic-archive.com, accessed on 7 August 2023), the Breast Ultrasound Images (BUSI) database (https://scholar.cu.edu.eg, accessed on 8 August 2023) and the Multi-Organ Nucleus Segmentation Challenge (MoNuSeg) database (https://monuseg.grand-challenge.org, accessed on 9 August 2023). Moreover, data supporting the findings of this study are available from the corresponding authors upon reasonable request.

## References

[1] Gubala V., Harris L.F., Ricco A.J., Tan M.X., Williams D.E. (2012). Point of care diagnostics: Status and future. Anal. Chem..

[2] Wang C., Liu M., Wang Z., Li S., Deng Y., He N. (2021). Point-of-care diagnostics for infectious diseases: From methods to devices. Nano Today.

[3] CDC Homepage. https://www.cdc.gov.

[4] Valanarasu J.M.J., Patel V.M. (2022). Unext: Mlp-based rapid medical image segmentation network. Proceedings of the International Conference on Medical Image Computing and Computer-Assisted Intervention.

[5] Codella N., Rotemberg V., Tschandl P., Celebi M.E., Dusza S., Gutman D., Helba B., Kalloo A., Liopyris K., Marchetti M. (2019). Skin lesion analysis toward melanoma detection 2018: A challenge hosted by the international skin imaging collaboration (isic). arXiv.

[6] Tschandl P., Rosendahl C., Kittler H. (2018). The HAM10000 dataset, a large collection of multi-source dermatoscopic images of common pigmented skin lesions. Sci. Data.

[7] Al-Dhabyani W., Gomaa M., Khaled H., Fahmy A. (2020). Dataset of breast ultrasound images. Data Brief.

[8] He K., Zhang X., Ren S., Sun J. (2015). Spatial pyramid pooling in deep convolutional networks for visual recognition. IEEE Trans. Pattern Anal. Mach. Intell..

[9] Fu Y., Lei Y., Wang T., Curran W.J., Liu T., Yang X. (2021). A review of deep learning based methods for medical image multi-organ segmentation. Phys. Med..

[10] Moorthy J., Gandhi U.D. (2022). A Survey on Medical Image Segmentation Based on Deep Learning Techniques. Big Data Cogn. Comput..

[11] Krizhevsky A., Sutskever I., Hinton G.E. (2012). Imagenet classification with deep convolutional neural networks. Adv. Neural Inf. Process. Syst..

[12] Vaswani A., Shazeer N., Parmar N., Uszkoreit J., Jones L., Gomez A.N., Kaiser Ł., Polosukhin I. (2017). Attention is all you need. Adv. Neural Inf. Process. Syst..

[13] Long J., Shelhamer E., Darrell T. Fully convolutional networks for semantic segmentation. Proceedings of the IEEE Conference on Computer Vision and Pattern Recognition.

[14] Ronneberger O., Fischer P., Brox T. (2015). U-net: Convolutional networks for biomedical image segmentation. Proceedings of the Medical Image Computing and Computer-Assisted Intervention–MICCAI 2015: 18th International Conference.

[15] Zhou Z., Rahman Siddiquee M.M., Tajbakhsh N., Liang J. (2018). Unet++: A nested u-net architecture for medical image segmentation. Proceedings of the Deep Learning in Medical Image Analysis and Multimodal Learning for Clinical Decision Support: 4th International Workshop, DLMIA 2018, and 8th International Workshop, ML-CDS 2018, Held in Conjunction with MICCAI 2018.

[16] Zhang Z., Liu Q., Wang Y. (2018). Road extraction by deep residual u-net. IEEE Geosci. Remote Sens. Lett..

[17] Huang H., Lin L., Tong R., Hu H., Zhang Q., Iwamoto Y., Han X., Chen Y.W., Wu J. Unet 3+: A full-scale connected unet for medical image segmentation. Proceedings of the ICASSP 2020—2020 IEEE international Conference on Acoustics, Speech and Signal Processing (ICASSP).

[18] Hasan M.K., Dahal L., Samarakoon P.N., Tushar F.I., Martí R. (2020). DSNet: Automatic dermoscopic skin lesion segmentation. Comput. Biol. Med..

[19] Tang P., Liang Q., Yan X., Xiang S., Sun W., Zhang D., Coppola G. (2019). Efficient skin lesion segmentation using separable-Unet with stochastic weight averaging. Comput. Methods Programs Biomed..

[20] Raffel C., Shazeer N., Roberts A., Lee K., Narang S., Matena M., Zhou Y., Li W., Liu P.J. (2020). Exploring the limits of transfer learning with a unified text-to-text transformer. J. Mach. Learn. Res..

[21] Lauriola I., Lavelli A., Aiolli F. (2022). An introduction to deep learning in natural language processing: Models, techniques, and tools. Neurocomputing.

[22] Chen L.C., Zhu Y., Papandreou G., Schroff F., Adam H. Encoder-decoder with atrous separable convolution for semantic image segmentation. Proceedings of the European conference on computer vision (ECCV).

[23] Chen J., Lu Y., Yu Q., Luo X., Adeli E., Wang Y., Lu L., Yuille A.L., Zhou Y. (2021). Transunet: Transformers make strong encoders for medical image segmentation. arXiv.

[24] Valanarasu J.M.J., Oza P., Hacihaliloglu I., Patel V.M. (2021). Medical transformer: Gated axial-attention for medical image segmentation. Proceedings of the Medical Image Computing and Computer Assisted Intervention–MICCAI 2021: 24th International Conference.

[25] Wang W., Chen C., Ding M., Yu H., Zha S., Li J. (2021). Transbts: Multimodal brain tumor segmentation using transformer. Proceedings of the Medical Image Computing and Computer Assisted Intervention–MICCAI 2021: 24th International Conference.

[26] Hatamizadeh A., Tang Y., Nath V., Yang D., Myronenko A., Landman B., Roth H.R., Xu D. Unetr: Transformers for 3d medical image segmentation. Proceedings of the IEEE/CVF Winter Conference on Applications of Computer Vision.

[27] Xu G., Li J., Gao G., Lu H., Yang J., Yue D. (2023). Lightweight Real-Time Semantic Segmentation Network With Efficient Transformer and CNN. IEEE Trans. Intell. Transp. Syst..

[28] Yuan F., Zhang Z., Fang Z. (2023). An effective CNN and Transformer complementary network for medical image segmentation. Pattern Recognit..

[29] Li Z., Zheng Y., Shan D., Yang S., Li Q., Wang B., Zhang Y., Hong Q., Shen D. Scribformer: Transformer makes cnn work better for scribble-based medical image segmentation. IEEE Trans. Med. Imaging.

[30] Cao H., Wang Y., Chen J., Jiang D., Zhang X., Tian Q., Wang M. (2022). Swin-unet: Unet-like pure transformer for medical image segmentation. Proceedings of the European Conference on Computer Vision.

[31] Zhang X., Cao X., Wang J., Wan L. (2023). G-UNeXt: A lightweight MLP-based network for reducing semantic gap in medical image segmentation. Multimed. Syst..

[32] Chan S., Huang C., Bai C., Ding W., Chen S. (2022). Res2-UNeXt: A novel deep learning framework for few-shot cell image segmentation. Multimed. Tools Appl..

[33] Xie E., Wang W., Yu Z., Anandkumar A., Alvarez J.M., Luo P. (2021). SegFormer: Simple and efficient design for semantic segmentation with transformers. Adv. Neural Inf. Process. Syst..

[34] Wang C.Y., Bochkovskiy A., Liao H.Y.M. YOLOv7: Trainable bag-of-freebies sets new state-of-the-art for real-time object detectors. Proceedings of the IEEE/CVF Conference on Computer Vision and Pattern Recognition.

[35] Yu Z., Yu L., Zheng W., Wang S. (2023). EIU-Net: Enhanced feature extraction and improved skip connections in U-Net for skin lesion segmentation. Comput. Biol. Med..

[36] Kumar N., Verma R., Anand D., Zhou Y., Onder O.F., Tsougenis E., Chen H., Heng P.A., Li J., Hu Z. (2019). A multi-organ nucleus segmentation challenge. IEEE Trans. Med. Imaging.

[37] Kumar N., Verma R., Sharma S., Bhargava S., Vahadane A., Sethi A. (2017). A dataset and a technique for generalized nuclear segmentation for computational pathology. IEEE Trans. Med. Imaging.

